# Cyber gaming addiction and impulsivity in adolescents

**DOI:** 10.1192/j.eurpsy.2023.1085

**Published:** 2023-07-19

**Authors:** K. Chiha, K. Khemakhem, M. Chaabane, D. Ben Touhemi, W. Kammoun, J. Boudabous, I. HadjKacem, H. Ayadi, Y. Moalla

**Affiliations:** Child Psychiatry, Hedi Chaker University Hospital, Sfax, Tunisia

## Abstract

**Introduction:**

The relationship between cyber video game addiction and impulsivity is controversial. Some studies have shown a significant link, others have found no association between the two behaviours.

**Objectives:**

To study impulsivity in adolescents with problematic use of internet video games.

**Methods:**

This was a cross-sectional, descriptive and analytical study, conducted among a sample of adolescents randomly collected in 6 schools in the region of Sfax-Tunisia, during the month of February 2022. The rate of addiction to video games was assessed by the 20-item “Internet Gaming Disorder-20” (IGD-20) scale and impulsivity by the 30-item “Barratt Impulsivity Scale” (BIS-11). Both scales are validated in Arabic.

**Results:**

The study involved 360 secondary school students, with a mean age of 16.62 +/- 0.822 years. The sex ratio was 1.09.

A gaming addiction was found in 4.7% of cases.

Similarly, impulse control disorder was noted in 23.6% of adolescents.

Problematic internet game use was significantly related to motor impulsivity (p=0.025).

There was no significant association between cyber video game addiction and cognitive or non-planning impulsivity.

**Conclusions:**

According to the results, impulsiveness is a factor to consider for understanding the development of addiction to internet video games. Thus, impulsiveness should be taken into account to explain problematic gaming behaviour as well as to design preventive and treatment interventions.

**Disclosure of Interest:**

None Declared

